# Surface chemistry of SnO_2_ nanowires on Ag-catalyst-covered Si substrate studied using XPS and TDS methods

**DOI:** 10.1186/1556-276X-9-43

**Published:** 2014-01-25

**Authors:** Michal Sitarz, Monika Kwoka, Elisabetta Comini, Dario Zappa, Jacek Szuber

**Affiliations:** 1 Institute of Electronics, Silesian University of Technology, Gliwice, 44-100, Poland; 2 SENSOR Lab, Department of Information Engineering, Brescia University and CNR IDASC, Brescia, 25133, Italy

**Keywords:** SnO_2_, Nanowires, Ag, Surface chemistry, XPS, TDS, Surface morphology, SEM

## Abstract

In this paper we investigate the surface chemistry, including surface contaminations, of SnO_2_ nanowires deposited on Ag-covered Si substrate by vapor phase deposition (VPD), thanks to x-ray photoelectron spectroscopy (XPS) in combination with thermal desorption spectroscopy (TDS). Air-exposed SnO_2_ nanowires are slightly non-stoichiometric, and a huge amount of C contaminations is observed at their surface. After the thermal physical desorption (TPD) process, SnO_2_ nanowires become almost stoichiometric without any surface C contaminations. This is probably related to the fact that C contaminations, as well as residual gases from air, are weakly bounded to the crystalline SnO_2_ nanowires and can be easily removed from their surface. The obtained results gave us insight on the interpretation of the aging effect of SnO_2_ nanowires that is of great importance for their potential application in the development of novel chemical nanosensor devices.

## Background

In the last two decades, tin dioxide (SnO_2_) has attracted a great interest because of its potential application for resistivity-type gas sensor devices. This is related to both high electric conductivity (approximately 10^2^ Ω^-1^ ·cm^-1^), compatible with standard electronics, and to the fact that the surface is chemically very active, in the presence of oxidizing and reducing gases [1-3].

Among SnO_2_ solid state gas sensor devices, those employing thin film technology are the most promising in terms of gas sensing response [4], stability, sensitivity, and especially compatibility with the downscaling of the electronic devices [5,6]. However, both thick and thin film performances are limited by the extension of active surface that potentially reduces their sensitivity. Nowadays, the research is focusing on nanostructured materials, like nanowires, nanorods, nanotubes, and nanoribbons [7,8] because they have a large surface-to-volume ratio and show enhanced chemical stability [9,10] and electrical performances [11]. Nanowires probably present the most interesting morphology for the fabrication of gas sensing devices, having about 30% atoms that are localized just at the surface, where the sensor transduction mechanism takes place [12,13], and thus enhancing the sensitivity. This is why SnO_2_ nanowires seem to be an interesting active material for gas sensor nanometer-scaled devices.

Another critical problem concerning the SnO_2_ thin films is the aging effect after their exposure to the surrounding atmosphere, which is related to undesired and uncontrolled adsorption of some contaminants at their surface, especially native oxide containing various C carbon species [14]. Even worse, this undesired adsorption cannot be avoided, since air atmosphere is the natural working condition for gas sensors. Nanowires may present slightly different behaviors compared to their polycrystalline counterparts and it is important to investigate their surface and surface-environment interaction for their possible integration as reliable sensors.

In this paper we present the results of experimental studies performed on SnO_2_ nanowires, prepared by vapor phase deposition (VPD) method on the Ag-covered Si substrate. We used x-ray photoelectron spectroscopy (XPS) in combination with thermal desorption spectroscopy (TDS) to investigate the surface of samples in air atmosphere. The obtained information have been interpreted on the base of the surface morphology, additionally checked by the scanning electron microscope (SEM).

## Methods

SnO_2_ nanowires were synthetized at SENSOR Lab, Department of Information Engineering, Brescia University, Italy, and Si (100) wafers have been used as substrates. Firstly, we deposited an ultrathin (5 nm) Ag nanolayers on the Si (100) substrate by RF magnetron sputtering (Kenotec Sputtering System, 50 W argon plasma, RT, 5 × 10^-1^ Pa, 7 sccm Ar flow). This ultrathin Ag layer plays an important role, promoting nucleation sites during the deposition process of SnO_2_ nanowires on the Si (100) substrate. SnO_2_ nanowires were then synthetized on Si (100) substrates by VPD in an alumina tubular furnace (custom design, based on a Lenton furnace). SnO_2_ powder (Sigma-Aldrich Corporation, St. Louis, MO, USA) was used as a source material for the deposition. It was placed in the middle of the furnace on an alumina crucible and heated up to 1,370°C to induce evaporation. Ag-covered Si (100) substrates were placed in a colder region of the furnace. Argon was used as gas carrier to achieve a significant mass transport towards the substrates. As the evaporated material reaches the colder region, it condensates on the substrates, forming SnO_2_ nanowires. The pressure inside the alumina tube was kept at 100 mbar, while the Ag-covered Si (100) substrates were kept at a temperature of 850°C.

The surface morphology of deposited SnO_2_ nanowires was examined using SEM (Zeiss, Leo 1525 Gemini model; Carl Zeiss AG, Oberkochen, Germany) at SENSOR Lab to confirm the proper synthesis of the nanostructures.

The fabricated nanostructures were then exposed to environmental atmosphere. The surface chemistry, including contaminations, of the obtained SnO_2_ nanowires was checked by XPS method. These experiments were performed at CESIS Centre, Institute of Electronics, Silesian University of Technology, Gliwice, Poland, using a XPS spectrometer (SPECS) equipped with the x-ray lamp (AlKα, 1,486.6 eV, XR-50 model), and a concentric hemispherical analyzer (PHOIBOS-100 model; SPECS Surface Nano Analysis GmbH, Berlin, Germany). The basic working pressure was at the level of approximately 10^-9^ hPa. Other experimental details have been described elsewhere [15].

In order to obtain additional information on the behavior of surface contaminations after air exposure, subsequent TDS experiments have been performed. A residual gas analyzer (Stanford RGA100 model; Stanford Research Institute, Sunnyvale, CA, USA) and sample temperature programmable control unit (Dual Regulated Power Supply OmniVac-PS 120 Model) were used to perform the TDS analysis. During the thermal physical desorption (TPD) cycle, the TDS spectra of selected gases like H_2_, H_2_O, O_2_, and CO_2_ have been registered. Heating ramp was set at 6°C per minute, in the range of 50 to 350°C. Other experimental details have been described elsewhere [14].

## Results and discussion

**Figure 1:**
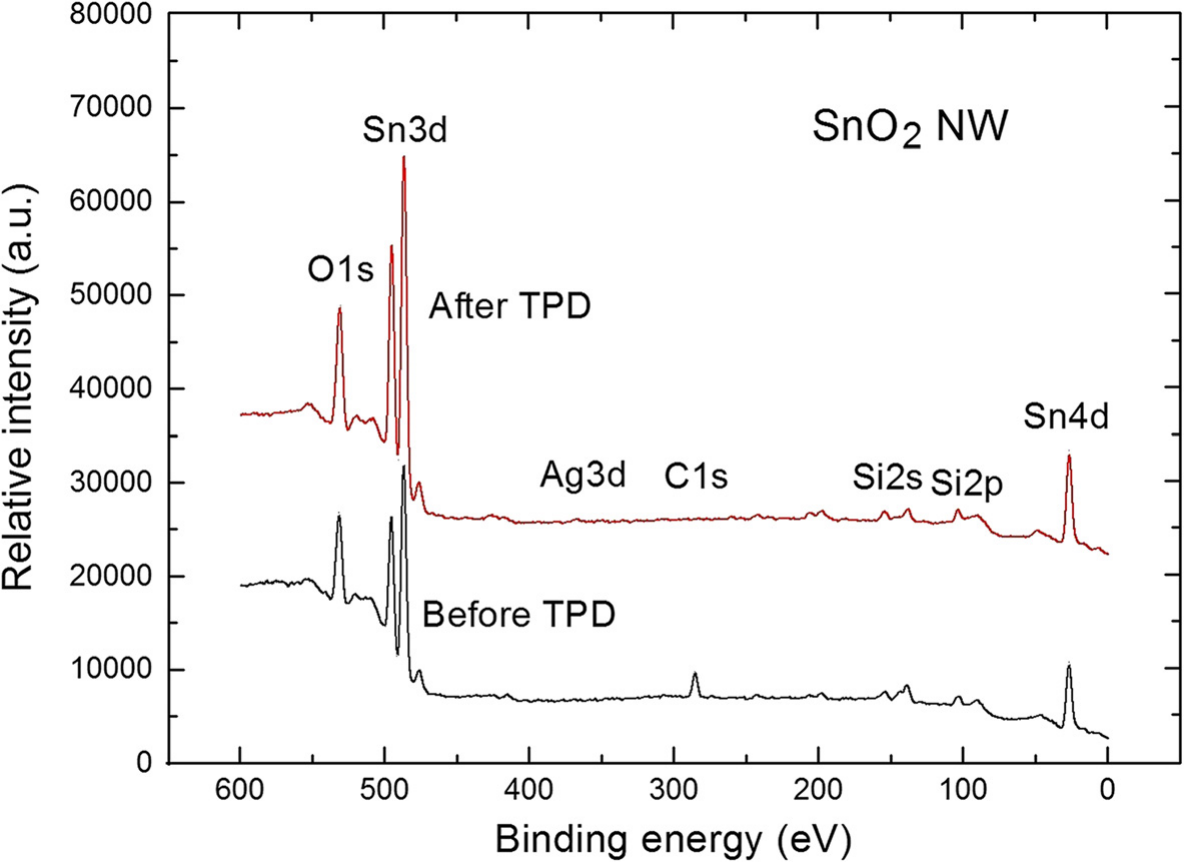
**XPS survey spectra of air-exposed SnO**_**2 **_**nanowires (before TPD process) and after subsequent TPD process.**

XPS and TDS comparative studies provide interesting information on the surface chemistry, including the behavior of surface contamination, of synthetized SnO_2_ nanowires. Figure 1 (lower part) shows the XPS survey spectrum of the VPD-deposited SnO_2_ nanowires after their preparation and exposure to air and before the TPD process. The spectrum contains the well-recognized main core level of XPS O1*s*, double Sn3*d*, and Sn4*d* peaks. Moreover, there is an evident contribution from the C1*s* peak related to strong surface carbon contamination. In turn, there is no contribution of XPS Ag3*d* double peaks, and this can be explained by the fact that the metal catalyst deposited at Si (100) substrate does not appear at the surface of grown SnO_2_ nanowires.

Quantitative analyses of surface chemistry (including stoichiometry) of SnO_2_ nanowires after air exposure have been performed. It consists in the determination of the relative concentration of the main components (within the escape depth of inelastic mean free path of photoelectrons of approximately 3 nm), based on the area (intensity) of the main core level XPS O1*s*, Sn3*d*, and C1*s*, weighted by the corresponding atomic sensitivity factor (ASF) [16]. The details of this procedure were already described in reference [14].

**Figure 2:**
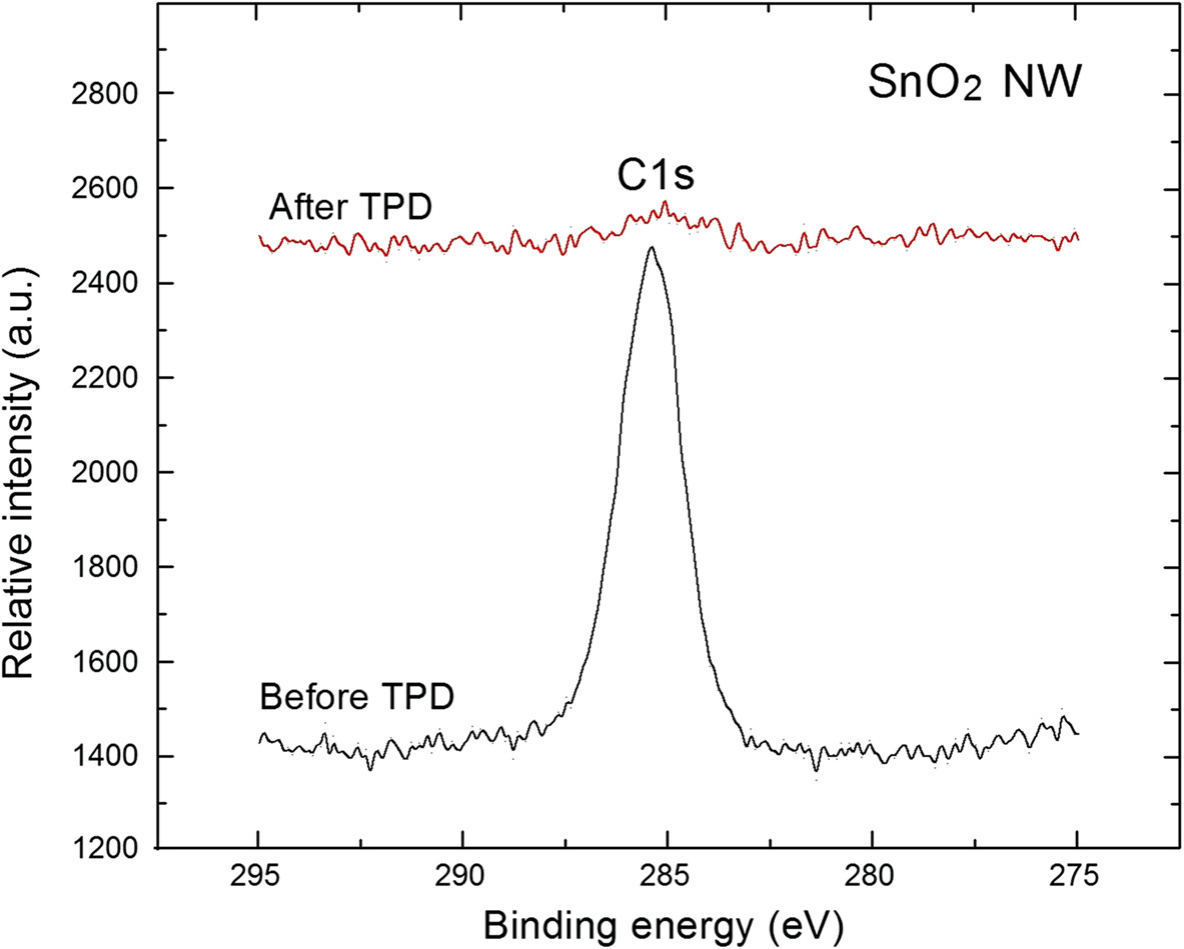
**XPS C1s spectra of air-exposed SnO**_**2 **_**nanowires before and after subsequent TPD process.**

According to this analysis, the relative [O]/[Sn] concentration on the surface of SnO_2_ nanowires after air exposure, was about 1.55 ± 0.05. It means that these SnO_2_ nanowires are slightly non-stoichiometric. This is probably related to the presence of oxygen vacancy defects in the surface region of the SnO_2_ nanowires recently identified by Kar et al. [17-19] for the SnO_2_ nanowires prepared by vapor-liquid-solid method with rapid thermal annealing from the UV photoluminescence (PL) measurements in combination with XPS, Raman, and transmission electron microscopy (TEM) studies. Probably, these oxygen vacancies can be treated as the surface active center responsible for the strong adsorption of different C species (contaminations) of the air-exposed SnO_2_ nanowires, what was confirmed by the corresponding relative [C]/[Sn] concentration estimated as 2.30 ± 0.05. This is additionally indicated by the XPS C1*s* spectrum shown in Figure 2 (lower spectrum).

The surface chemistry, including C contamination, of the SnO_2_ nanowires was evidently changed after subsequent TPD process, as shown in the corresponding XPS survey spectrum (Figure 1, higher line).

Firstly, the relative [O]/[Sn] concentration increased, reaching a value of 1.75 ± 0.05, corresponding to the improvement of their stoichiometry. Moreover, there is no evident contribution from the XPS C1*s*, which means that, during the TPD process, the undesired C contaminations from the air atmosphere, found on the surface of SnO_2_ nanowires, were removed. This corresponds to the almost complete vanishing of XPS C1*s* peak shown in Figure 2 (higher spectrum). These last observations, i.e. that C contamination from the surface of SnO_2_ nanowires can be easily removed by the vacuum thermal treatment, are of great importance for their potential application as gas sensors material. This point will be more precisely addressed later on.

**Figure 3:**
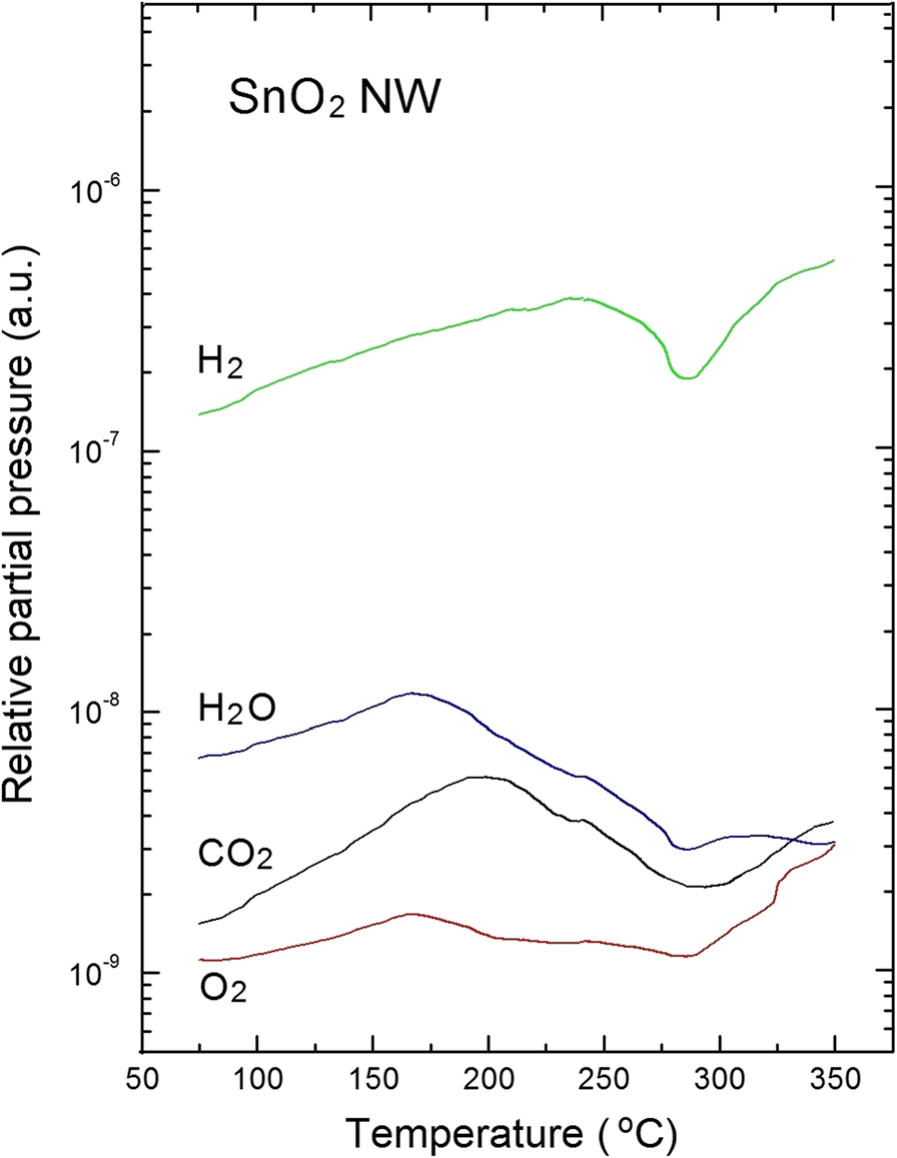
**TDS spectra of main residual gases desorbed from the SnO**_**2 **_**nanowires exposed to air.**

Moreover, it should be pointed out that after the TPD process there is no contribution of XPS Ag3*d*, which means that, similarly to untreated SnO_2_ nanowires, Ag is not observed at the surface of SnO_2_ nanowires even after TPD process. Ag catalyst probably remains on the silicon substrate. It surely plays a significant role in inducing the nucleation of the nanowires on the substrates, however it may not have some significant effects on the sensing performances of tin dioxide nanowires. This is the main reason of our choice to use Ag as catalyst instead of Au nanoparticles. It has been demonstrated that SnO_2_ nanowires have a Au nanoparticle on the tip [20]. This could affect the sensing performances of devices fabricated using tin dioxide nanowires as sensing elements. We use Ag as growth catalyst to prevent possible catalytic effects of the metal particle during the gas sensing measurements. All obtained information on the evolution of SnO_2_ nanowires surface chemistry before and after TPD process are in a good correlation with the respective TDS spectra shown in Figure 3. The registered TDS spectra have been corrected by the ionization probability of respected gases detected in our experiments.

From the TDS spectra shown in Figure 3 one can easily note that only small amount of the molecular oxygen (O_2_) desorbs from the SnO_2_ nanowires already at the relative partial pressure of about 10^-9^ mbar at 170°C approximately. The molecular hydrogen (H_2_) was desorbed during TPD process with highest relative partial pressure of about 10^-7^ mbar with a maximum at higher temperatures (approximately 260°C). These last observations are probably related to the high degree of crystallinity of SnO_2_ nanowires [21]. The molecular hydrogen seems not able to penetrate deeply the subsurface space. This experimental evidence has never been reported to the best of our knowledge. In turn, one can easily note that only small amount of the molecular oxygen (O_2_) desorbs from the VPD SnO_2_ nanowires already at the relative partial pressure of about 10^-9^ mbar at 170°C approximately. It means that probably the small amount of residual oxygen is only weakly (physically) bounded at the surface of SnO_2_ nanowires. It corresponds to a small increase of relative [O]/[Sn] concentration after TDS process, as evidenced from XPS measurements.

Concerning the case of water vapor (H_2_O), there is a maximum relative partial pressure of about 10^-8^ mbar at about 170°C, as can be seen from the respective TDS spectrum. This is quite similar to one of the molecular oxygen (O_2_) with a different value of maximum partial pressure (almost one order of magnitude higher).

The most important TPD effect was observed for carbon dioxide (CO_2_). The respective TDS spectrum exhibit a more complicated shape with two evident peaks: a wider one, having a maximum of relative partial pressure of about 10^-9^ mbar at about 200°C, and a narrow one, having a maximum partial pressure slightly smaller at about 350°C. It probably means that C containing surface contaminations is bounded in two different forms and with different bonding energy at the external surface of crystalline SnO_2_ nanowires.

These last observations related to the desorption behavior of water vapor (H_2_O) and carbon dioxide (CO_2_) were in a good correlation with an evident increase of relative [O]/[Sn] concentration, as well as almost complete vanishing C contaminations from the nanowires under investigations as determined by the XPS experiments. Thanks to the complete removal of C contaminations during TPD process the surface of SnO_2_ nanowires became almost stoichiometric, in a good agreement to the published electron diffraction data [22].

Additionally, TEM analysis [20,23] of SnO_2_ nanowires showed that these one-dimensional nanostructures are single crystals with atomically sharp terminations. They have the SnO_2_ cassiterite structure and grow along the [101] direction.

**Figure 4:**
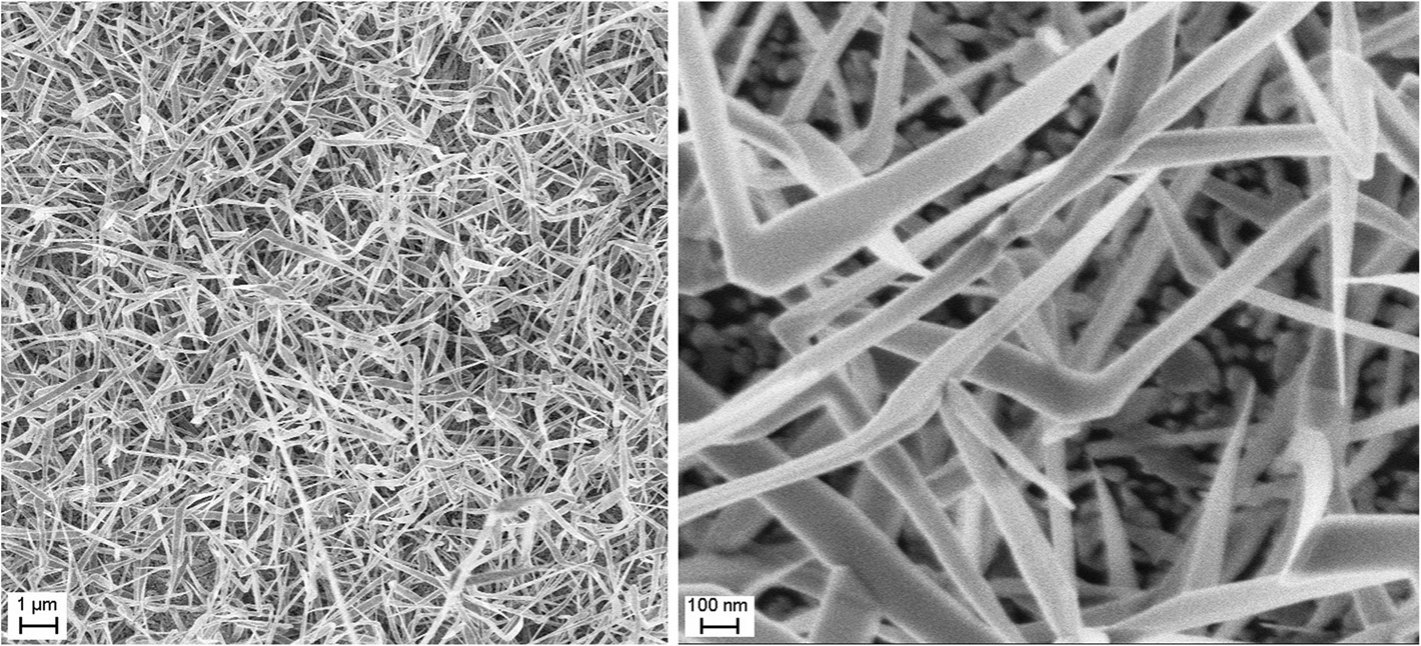
**SEM images of SnO**_**2 **_**nanowires of different magnification.**

The SEM images in Figure 4 report the morphology of SnO_2_ nanowires. Moreover, it is easy to estimate that the ratio between their length (several microns) and width (less than 100 nm) is very high.

All information reported above are crucial for potential application of SnO_2_ nanowires in the detection of C containing species. The last one, i.e., that there is a possibility to complete removal of C contaminations during TPD process from the surface of SnO_2_ nanowires, is of great importance because it allows to get shorter response/recovery time for the gas sensors systems based on SnO_2_ nanowires. This is in evident contradiction to the observation for the SnO_2_ thin films, as summarized in [5].

## Conclusions

SnO_2_ nanowires have been synthetized on Ag-covered Si (100) substrate by VPD technique. XPS and TDS were performed on the samples in order to understand the surface composition and the presence of carbon contaminations. XPS and TDS studies showed that SnO_2_ nanowires in the presence of air at atmospheric pressure are slightly non-stoichiometric, what was related to the presence of oxygen vacancy defects in their surface region. These oxygen vacancies are probably responsible for the strong adsorption (contamination) by C species of the air-exposed SnO_2_ nanowires.

After TPD process, SnO_2_ nanowires become almost stoichiometric without any surface carbon contamination, probably thanks to the fact that carbon contaminations, as well as residual gases from the air, are weakly bounded to the crystalline SnO_2_ nanowires and can be easily removed from their surface i.e., by thermal treatments.

These observations are of great importance for potential application of SnO_2_ nanostructures (including nanowires) in the development of gas sensor devices. They exhibit evidently better dynamics sensing parameters, like short response time and recovery time to nitrogen dioxide NO_2_, as observed in our recent studies [24].

## Abbreviations

NW: nanowires; SEM: scanning electron microscopy; TDS: thermal desorption spectroscopy; TEM: transmission electron microscopy; TPD: thermal physical desorption; VPD: vapor phase deposition; XPS: x-ray photoelectron spectroscopy

## Competing interests

The authors declare that they have no competing interests.

## Authors’ contributions

MS was involved in the preparation of samples, carrying out the SEM study, and engaged in XPS and TDS experiments and data analysis. MK carried out the XPS and TDS experiments, analyzed the experimental data, and drafted the manuscript. EC and JS conceived of the study. DZ was involved in the preparation of samples. All authors read and approved the final version of the manuscript.
